# Association of mTORC1‑dependent circulating protein levels with cataract formation: a mendelian randomization study

**DOI:** 10.1186/s12864-022-08925-7

**Published:** 2022-10-21

**Authors:** Yingjun Cai, Kangcheng Liu, Pengfei Wu, Ruolan Yuan, Fei He, Jing Zou

**Affiliations:** 1 Eye Center of Xiangya Hospital, Hunan Key Laboratory of Ophthalmology, Central South University, 410008 Changsha, Hunan China; 2 National Clinical Research Center for Geriatric Disorders, Xiangya Hospital, Central South University, 410008 Changsha, Hunan China; 3 Jiangxi Clinical Research center for Ophthalmic Disease, Jiangxi Research Institute of Ophthalmology and Visual Science, Affiliated Eye Hospital of Nanchang University, Nanchang, China; 4 Hunan Key Laboratory of Medical Genetics, School of Life Sciences, Central South University, Changsha, China; 5 The First Affiliated Hospital of Nanchang University, Nanchang, China

**Keywords:** mTOR, EIF4EBP, Circulating, Cataract, Mendelian randomization

## Abstract

**Background:**

The mechanistic target of rapamycin (mTOR) signal pathway plays a critical regulating role in the occurrence and development of cataract. However, the role of mTORC1 downstream proteins, including ribosomal protein S6K (RP-S6K), eukaryotic initiation factor 4E-binding protein (EIF4EBP), eukaryotic initiation factor 4G (EIF-4G), eukaryotic initiation factor 4E (EIF-4E), and eukaryotic initiation factor 4A (EIF-4A), in regulating cataract development is still unknown. Herein, we conducted a mendelian randomization (MR) study to understand the function of mTORC1 signaling in the process of cataract development.

**Results:**

The causal estimate was evaluated with inverse-variance weighted (IVW) estimate, weighted median estimator, MR-Egger and MR robust adjusted profile score (MR. RAPS). The single-nucleotide polymorphisms (SNPs), *P*<5 × 10^− 6^ and r^2^<0.05, were selected to genetically predict the RP-S6K, EIF4EBP, EIF-4E, EIF-4A, and EIF-4G. We included a total of 26,758 cases and 189,604 controls in this MR study. The study revealed causal association between circulating EIF4EBP (OR 1.09, 95% confidence interval 1.03,1.16, *P* = 0.004), RP-S6K (OR 1.04, 95% confidence interval 1.01, 1.08, *P* = 0.02) and cataract formation with IVW estimate. Whereas after correcting outliers, MR robust adjusted profile score (MR. RAPS) shows consistent result with IVW for EIF4EBP (OR = 1.08, 95%CI:1.05–1.11, *P* = 0.007). The observation strengthened the confidence in the true causal associations. However, no association was found for circulating EIF-4E (OR 1.03, 95% confidence interval 0.97, 1.09, *P* = 0.31), EIF-4A (OR 1.02, 95% confidence interval 0.98, 1.07, *P* = 0.34), and EIF-4G (OR 1.02, 95% confidence interval 0.94, 1.01, *P* = 0.64) levels with cataract formation. No evidence of heterogeneity and unbalanced horizontal pleiotropy was detected.

**Conclusion:**

The MR study suggests that EIF4EBP is a high-risk factor for cataract development. There may be a potential causal association between the mTORC1/EIF4EBP axis and cataract. This research highlights the potential mechanism for cataract development and a genetic target to prevent as well as treat cataracts.

**Supplementary Information:**

The online version contains supplementary material available at 10.1186/s12864-022-08925-7.

## Introduction

Cataract is one of the common causes of visual impairment and blindness in developing and developed countries [1–3]. Although the phacoemulsification application is available worldwide, over 12 million people are blind because of cataract until 2010 [1, 4]. Moreover, no effective pharmacological strategy exists to prevent the development of cataract. Several signaling molecules and pathways take part in modulating the development of cataract, including the mechanistic target of rapamycin (mTOR) [5], endoplasmic reticulum stress[6], and advanced glycation end products [7] et al. Of note, the mTOR signal pathway plays a critical regulating role in the occurrence and development of cataract by regulating cellar processes such as cell growth, autophagy, and epithelial-mesenchymal transition (EMT) [8–10].

**Fig. 1:**
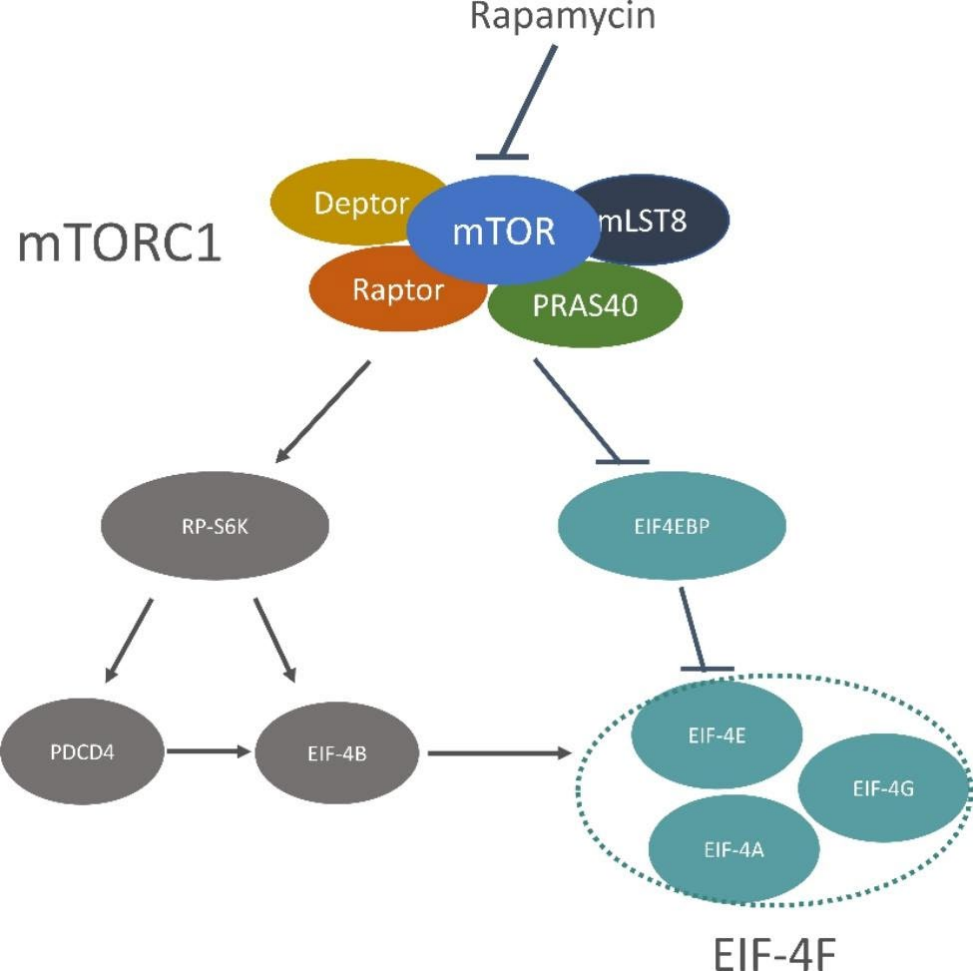
Main protein of mTORC1 signal pathway (mTORC1 is comprised by mTOR, Deptor, mLST8, Raptor, PRAS40. mTORC1 regulates cell function almost through phosphorylating the downstream protein of mTORC1, including PR-S6K and EIF4EBP. After getting phosphorylated by mTORC1, PR-S6K activates EIF-4B, which positively regulates EIF-4F. RP-S6K also phosphorylates and promotes the degradation of PDCD4, which is the inhibitor of EIF-4B. EIF4EBP is a repressor of EIF-4E, once phosphorylated by mTORC1, it dissociate with EIF-4E, and then promotes the assembly of EIF-4F complex. The EIF-4F complex is composed by EIF-4E, EIF-4G, and EIF-4A. The specificity repressor of mTORC1-rapamycin can combine with mTOR, and induce conformational change in mTORC1. RP-S6K, ribosomal protein S6K kinase; EIF4EBP, eukaryotic initiation factor 4E-binding protein; EIF-4G, translation initiation factor 4G; EIF-4E, translation initiation factor 4E; EIF-4A, translation initiation factor 4A)

In mammals, mTOR can combine with different proteins and generate different complexes, including the mechanistic target of rapamycin complex1 (mTORC1) and the mechanistic target of rapamycin complex2 (mTORC2) [11]. mTORC1 plays an important role in cellular senescence and anabolic processes, such as inhibiting autophagy, inducing apoptosis, regulating translational regulators, promoting secreting or restraining proinflammatory mediators, and displaying high metabolic rate or caloric restriction [5, 12–17]. The regulation of mTORC1 functions is primarily through phosphorylating the downstream targets, including ribosomal protein S6K kinase 1 (PR-S6K1) and eukaryotic initiation factor 4E-binding proteins (EIF4EBPs) (Fig. 1) [14]. After getting phosphorylated, PR-S6K1 activates eukaryotic initiation factor 4B (EIF-4B), which positively regulates the 5’cap binging translation eukaryotic initiation factor 4 F (EIF-4 F) [18]. RP-S6K1 also phosphorylates and promotes the degradation of PDCD4, which is the inhibitor of EIF-4B [19]. EIF4EBP is a repressor of eukaryotic translation initiation factor 4E (EIF-4E). Once phosphorylated by mTORC1 in multiple sites, it dissociates with EIF-4E, then promotes the 5’cap-dependent translation and assembling of the EIF-4F complex. The EIF-4F complex is composed of EIF-4E, EIF-4G, and eukaryotic initiation factor 4A (EIF-4A) (Fig. 1) [12].

Rapamycin binds with FKBP12, a part of mTOR, and forms a FKBP12-rapamycin complex (Fig. 1). Subsequently, the complex induces mTOR conformational change[20]. mTORC1 is sensitive to the nanomolar concentration of rapamycin within one hour of exposure, whereas mTORC2 requires a more prolonged exposure and a higher dose [21]. The change of conformation in mTOR causes the disruption between mTOR and Raptor in mTORC1 [21]. Meng et al. [8] observed that rapamycin can effectively impair EMT in lens epithelial cells, thus inhibiting the process of cataract. At the same time, Ping et al. [9] treated cataract zebrafish with rapamycin and discovered the mitigation effect of cataract. Considering the special role of rapamycin in regulating cataract, we speculated that mTORC1 signaling might affect cataract formation.

Mendelian randomization (MR) is an analytic approach for assessing the causal association between potentially modifiable exposures, defined as single-nucleotide polymorphisms (SNPs), and the clinically relevant outcome [22]. Compared with randomized controlled trials (RCTs)-the gold standard to establish the causal association, MR utilizes the data from large-scale genome-wide association studies (GWAS). Thus, MR study is more time-effective and contains a larger sample size. Moreover, sometimes RCTs cannot be conducted as they are costly, impartial, and even unethical[23]. In our study, we conducted a MR study by leveraging the extensive European population-based GWAS summary statistic data of mTORC1-related protein in plasma, including the concentration of RP-S6K, EIF4EBP, and the EIF-4F components, like EIF-4E, EIF-4A, EIF-4G. In addition, we also studied the causal effect of mTORC1‑related circulating protein levels on cataract formation.

## Method

### Study design and participant flowchart

**Fig. 2:**
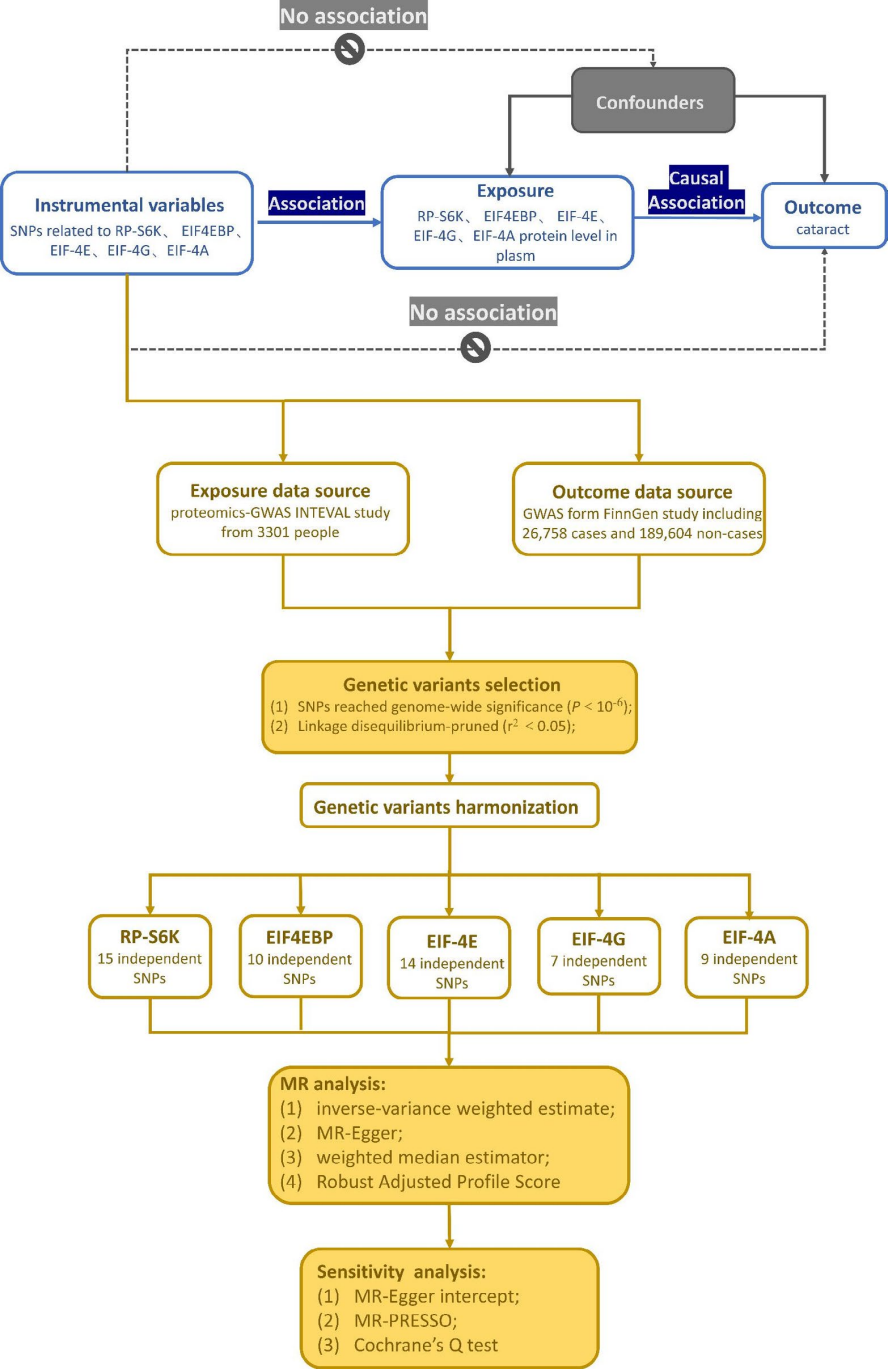
Key assumptions and flow chat of Mendelian randomization study (This MR study fulfilled the three core assumptions: **(1)** IVs must be associated with exposure, **(2)** genetic instrument must not be associated with confounders, **(3)** must be no association between genetic instruments and cataract except through exposure. SNP, single nucleotide polymorphism; RP-S6K, ribosomal protein S6K kinase; EIF4EBP, eukaryotic initiation factor 4E-binding protein; EIF-4G, translation initiation factor 4G; EIF-4E, translation initiation factor 4E; EIF-4A, translation initiation factor 4A.)

We conducted a two-sample MR study that relied on two different GWAS summary statistics. For the definition of exposures, we applied downstream targets of mTORC1 in plasma, like RP-S6K, EIF4EBP, and three components of EIF-4 F, like EIF-4E, EIF-4A, and EIF-4G. We also obtained related SNPs from the proteomics-GWAS INTEVAL study [24].We defined cataract as outcome, and for the outcome-associated SNPs, we leveraged the data from the FinnGen study. The flowchart and the incorporated sample size are shown in Fig. 2.

### Ethics

This study used published or publicly available summary data that did not involve primary study participants. Ethical approval of each study can be found in the original publications. These studies were conducted with the ethical guidelines of the 1975 Declaration of Helsinki.

### Data sources for MR analyses

#### Data sources for exposure

The genetic variants of mTORC1‑dependent protein in plasma were obtained from the publicly available proteomics-GWAS INTEVAL study (https://www.phpc.cam.ac.uk/ceu/proteins/), which included 3622 plasma proteins from 3301 healthy volunteers [24]. The INTEVAL study lasted from mid-2012 to mid-2014 and recruited blood donors over 18 years old from 25 centers of England’s National Health Service Blood and Transplant (NHSBT) [25]. The protein concentration was measured by a multiplexed, aptamer-based approach-Slow Off-rate Modified Aptamers (SOMAscan assay). This approach helps analyze lower detectable protein concentrations than traditional methods [26, 27]. It uses the modified single standard DNA SOMAmers to bind with protein targets quantified through DNA microarray [28]. The protein abundances were quantified as relative fluorescent units [24].

The genome-wide associations were obtained from the relative protein abundances after natural log-transformed, then adjusted for age, sex, time costing on blood collection, multi-dimensional scale according to population structural with linear regression, and genetic association[24]. To obtain sufficient SNPs and estimate the accurate result, we obtained genome-wide significantly (*P*<5 × 10^− 6^) and independently (R^2^<0.05) SNPs to predict mTORC1‑dependent protein in plasma [29–32], including RP-S6K, EIF4EBP, EIF-4G, EIF-4E, and EIF-4A, from the proteomics-GWAS INTEVAL study. To ensure the reliability of MR results, we removed SNPs with palindromes.

#### Data sources for the outcome

The association of outcome relative SNPs with cataract were obtained from the FinnGen study [33] (https://r5.finngen.fi/). This study defined cataract by H25 of the International Classification of Disease-10 (ICD-10). The genotype data of the FinnGen research project was from Finnish biobanks and digital health record data from Finnish health registries. The 5th data was released in the first quarter of 2020 with a total sample size of 260,405. The individuals with ambiguous gender, high genotype missingness (> 5%), excess heterozygosity (± 4SD), and non-Finnish ancestry were excluded. Finally, 26,758 cases and 189,604 controls of Finnish descent were included in this study.

### Assessment of assumptions

The MR study fulfilled the three core assumptions (Fig. 2) [34] :(1) The IVs must be associated with RP-S6K, EIF4EBP, EIF-4G, EIF-4E and EIF-4A. (2) The genetic instrument must not be associated with confounders, conditional on the exposure-outcome relationship. (3) There should be no association between genetic instruments and cataracts except through EIF-4E, EIF-4A, EIF-4G, EIF4EBP, and RP-S6K. We tested assumptions (2) (3) by the heterogeneity and pleiotropy test. We tested heterogeneity with Cochrane’s Q value. The pleiotropy was divided into vertical and horizontal pleiotropy according to the pathways which SNPs influence the traits[35]. Considering vertical pleiotropy never violates the MR assumption and does not cause bias[36], we only tested the horizontal pleiotropy with the MR Egger regression intercept and MR-PRESSO.

### Sensitivity analysis

To test and correct for horizontal pleiotropy and examine the robustness of the MR estimates, we applied the value of intercept in MR-Egger regression and mendelian randomization pleiotropy residual sum and outlier (MR-PRESSO) [37]. The intercept value in the MR-Egger regression was equivalent to the magnitude of unbalanced pleiotropic[38]. We also applied the MR-PRESSO to test and correct for the outlier in the inverse-variance weighted (IVW) estimate [37]. This approach included three components: global test, outlier test, and distortion test [37]. MR-PRESSO removes SNPs that were questioned and refitted other SNPs in the IVW regression, and then estimates the causal effect again [37]. The result of the causal estimate is unbiased even if outliers are up to 50% [37]. To test for the heterogeneity, we used Cochrane’s Q value. For the robustness of the MR estimate, we applied the leave-one-out approach to test whether any single SNPs drove the estimate. In the leave-one-out approach, all SNPs but one was calculated by IVW regression. If there is sufficient evidence for the causal association, the excluded SNP did not drive the MR results.

### Statistical method

“TwoSampleMR” packages were used to obtain the MR estimate in the R software (version1.4.1717). The SNPs were aligned by the allele letter and frequency. We used IVW to obtain the MR estimates. In case of no evidence for horizontal pleiotropy, and weak instruments, the IVW was used to obtain an overall estimate using a formula with the meta-analysis method of Wald estimates. The formula is the ratio of the gene‐outcome association and the gene‐exposure association of each exposure.[39] Weight median (WM) estimator[40] and MR-Egger [38, 41] were used to further analyze MR results. The WM estimator provided a consistent estimate when over 50% of the weight came from valid IVs [40]. Unlike IVW, the MR-Egger allows for little evidence of a violation of the IV3 assumption (instrumental variable assumption 3), which means permitting a low degree of evidence of the pleiotropic effects and still obtaining an unbiased causal estimate. However, it consumes statistical power [41]. Therefore, it is generally believed that the results of IVW are the most meaningful when passing the sensitivity analysis [42–44]. Robust Adjusted Profile Score (MR. RAPS) was calculated for the causal estimate to provide higher statistical power, despite the existence of many weak instrumental variables [45]. A circle map was used to visualize the result, which combined the MR estimate of all valid SNPs and the meta-analysis of MR estimate. The MR results were described by odds ratio (OR) and confidence interval (CI). *P values* < 0.05 were considered statistically significant.

## Results

### MR estimate of RP-S6K

**Fig. 3:**
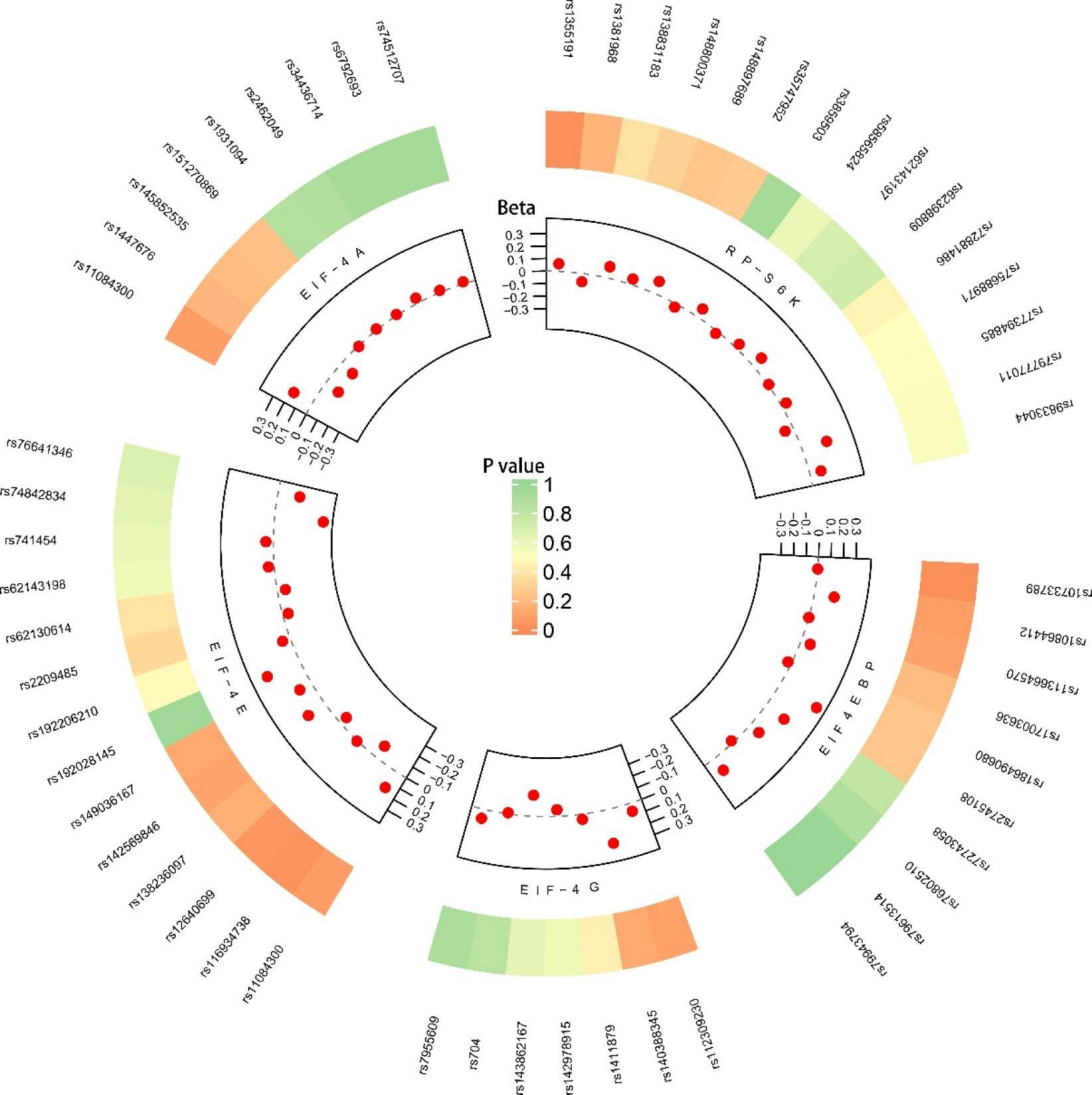
SNPs influence the causal-effect with MR estimate. (Each dots represents the causal effect on the result of each IV, and each region corresponds to a different mTORC1 downstream protein, including RP-S6K, EIF4EBP, EIF-4G, EIF-4E, and EIF-4A. The outer annular layer represents the *P-value* of MR, “*P-value* of MR” represent the *P-value* of the corresponding “beta value of each SNP”. The inner layer is the beta value of each SNP, beta coefficients are shown as per 1 standard deviation increase in the trait, and β = log(or). The gray dotted line between inner layer means beta equals zero. SNP, single nucleotide polymorphism; RP-S6K, ribosomal protein S6K kinase; EIF4EBP, eukaryotic initiation factor 4E-binding protein; EIF-4G, translation initiation factor 4G; EIF-4E, translation initiation factor 4E; EIF-4A, translation initiation factor 4A; MR, Mendelian randomization.)

After excluding SNPs having linkage disequilibrium, outline, or palindromic sequence, 15 SNPs were included to predict RP-S6K genetically (Supplementary Table 1). The overall estimate calculated with IVW revealed the higher genetically predicted RP-S6K significance causal association with cataract. However, after correcting outliers, MR. RAPS results showed no significant causal association. Additionally, MR-Egger and weight median showed no association between the RP-S6K and cataract (Table 1). All the SNPs which were genetically predicting RP-S6K are shown on Fig. 3.

**Table 1:** Mendelian Randomization (MR) estimate for the association between mTORC1/RP-S6K, mTORC1/EIF4EBP pathway, and cataract

Exposure	Method	OR (95% CI)	***P-value***
RP-S6K	IVW	1.04(1,01-1.08)	0.021
	WM	1.04(0.99–1.09)	0.103
	MR Egger	1.00(0.94–1.07)	0.974
	MR. RAPS	1.03(1.01–1.04)	0.113
EIF4EBP	IVW	1.09(1.03–1.16)	0.004
	WM	1.09(1.00-1.18)	0.038
	MR Egger	1.06(0.93–1.22)	0.391
	MR. RAPS	1.08(1.05–1.11)	0.007
EIF-4G	IVW	1.02(0.94–1.10)	0.643
	WM	1.02(0.94–1.13)	0.548
	MR Egger	1.07(0.88–1.29)	0.541
	MR. RAPS	1.04(1.00-1.08)	0.333
EIF-4E	IVW	1.03(0.97–1.09)	0.308
	WM	1.05(0.99–1.11)	0.107
	MR Egger	1.01(0.90–1.15)	0.816
	MR. RAPS	1.04(1.02–1.06)	0.066
EIF-4A	IVW	1.03(0.98–1.07)	0.338
	WM	1.03(0.97–1.08)	0.315
	MR Egger	1.03(0.95–1.12)	0.506
	MR. RAPS	1.02(1.00-1.05)	0.291

**Abbreviations**: OR odds ratio; RP-S6K, ribosomal protein S6K kinase; EIF4EBP, eukaryotic initiation factor 4E-binding protein; EIF-4G, translation initiation factor 4G; EIF-4E, translation initiation factor 4E; EIF-4A, translation initiation factor 4A; IVW, inverse-variance weighted estimate; WM, weighted median estimator

### MR estimate of EIF4EBP

After quality control, 10 SNPs were obtained to predict EIF4EBP (Supplementary Table 1). IVW showed that EIF4EBP was positively associated with cataract, and the result of MR. RAPS was similar (Table 1), which strengthened the confidence of the true causal associations[46]. Meanwhile, the association remained directionally consistent in weight median, whereas MR-Egger showed no association between mTORC1-dependent protein level of EIF4EBP with cataract (Table 1). All SNPs influenced by the causal-effect with MR are shown with a circle map (Fig. 3).

### MR estimate of EIF-4G, EIF-4E and EIF-4A

Similarly, we obtained 14 SNPs for EIF-4E, 9 SNPs for EIF-4A, and 7 SNPs for EIF-4G (Supplementary Table 1). The EIF-4F components, including EIF-4E, EIF-4A, and EIF-4G were not associated with cataract (Table 1). The result of MR. RAPS was also similar. The influence of EIF-4E, EIF-4A, and EIF-4G related SNPs on cataract are shown in Fig. 3.

### Sensitivity analyses result

**Fig. 4:**
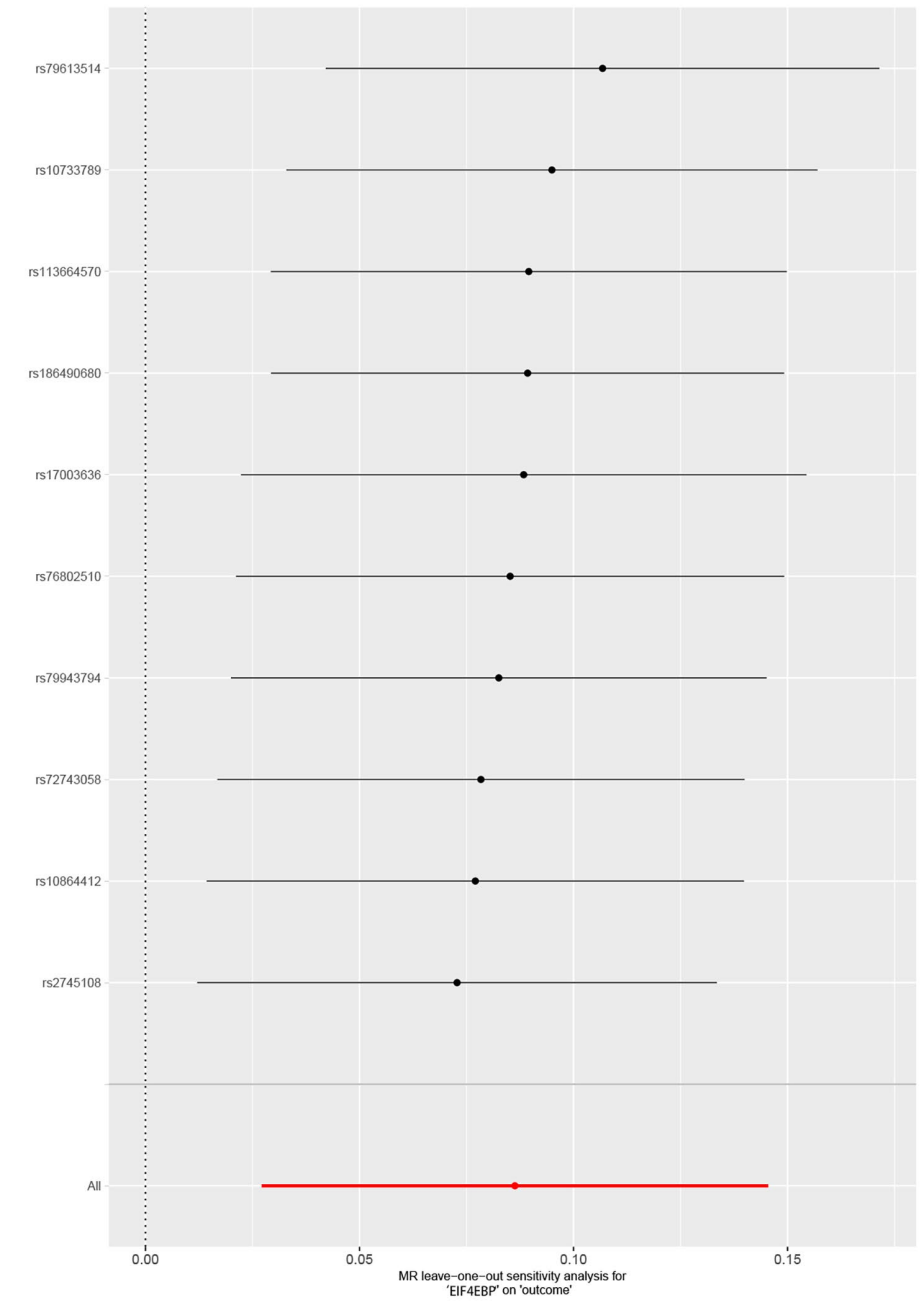
Leave-one-out analysis for the estimates of EIF4EBP on cataract. Leave-one-out result of EIF4EBP. The leave-one-out result shows that after excluding one SNP, the result of the MR estimate was almost stable, which was further verified by the robustness of MR results. EIF4EBP, eukaryotic initiation factor 4E-binding protein; MR, Mendelian randomization

All the sensitivity analyses result are presented in Table 2. The MR-Egger regression intercept showed insufficient evidence of unbalanced pleiotropy (all *P*>0.05). All the funnel plots were almost symmetrical confirming no sufficient evidence existed for pleiotropy (Supplementary Fig. 1). MR-PRESSO was further applied to test for the horizontal pleiotropy, which was not corrected by MR-Egger regression. The MP-PRESSO results of RP-S6K, EIF4EBP, EIF-4E, EIF-4A, and EIF-4G were similar (Table 2). Cochrane’s Q value tested the substantial heterogeneity statistics. Table 2 shows no heterogeneity for the SNPs that genetically predict circulating level of RP-S6K, EIF4EBP, EIF-4E, EIF-4A and EIF-4G. The leave-one-out method further verified the robustness of MR results (Fig. 4 and Supplementary Fig. 2). Considering there was no obvious evidence of unbalanced pleiotropy and heterogeneity with sensitivity analyses, IVW and MR. RAPS can be regarded as the main results of this study. Whereas after correcting the outlier with MR. RAPS, we deemed only EIF4EBP causally associated with cataract.

**Table 2:** Sensitivity analysis for the association between mTORC1 related protein and cataract

Exposure	Cochrane’s Q		Cochrane’s Q ***P-value***	MR-Egger Intercept	MR-Egger intercept ***P-value***	MR-PRESSO ***P-value***
RP-S6K		9.529	0.80	0.013	0.184	0.38
EIF4EBP		8.643	0.47	0.006	0.696	0.43
EIF-4G		6.378	0.38	-0.010	0.626	0.37
EIF-4E		21.201	0.07	0.004	0.806	0.49
EIF-4A		7.400	0.49	-0.002	0.838	0.49

**Abbreviations**: RP-S6K, ribosomal protein S6K kinase; EIF4EBP, eukaryotic initiation factor 4E-binding protein; EIF-4G, translation initiation factor 4G; EIF-4E, translation initiation factor 4E; EIF-4A, translation initiation factor 4A

## Discussion

This is the first study to assess the causal association of mTORC1-dependent plasma protein levels with cataract using the MR approach. With data from the large-scale GWAS of Finnish descent, our MR research is concerned with the causal association between mTORC1-related proteins, including RP-S6K, EIF4EBP, EIF-4E, EIF-4A, and EIF-4G and cataract. The MR study suggests the positive direct association between plasma protein level of EIF4EBP (OR = 1.09, 95% CI 1.03–1.16, *P* = 0.004) and the risk of cataract in a combined sample of 26,758 cases and 189,604 controls. However, this study provided no evidence for the association of genetically determined RP-S6K, EIF-4E, EIF-4A, and EIF-4G with the onset of cataract. Moreover, sensitivity analyses did not show sufficient evidence of unbalanced pleiotropy and heterogeneity, which indicates the MR estimate was independent and robust.

With aging, LECs undergo numerous biochemical and biophysical changes, which induces structural and functional impairment, leading to EMT development and autophagy activation[47]. Previous studies have indicated that mTORC1 possibly promotes the development of cataract through controlling cellular EMT and inhibiting the level of autophagy through related signaling proteins. However, the involvement of the downstream protein in the mTORC1 signal pathway is not proven. As a critical process in cataract development, EMT is responsible for the trans-differentiation of lens epithelial cells (LECs) into mesenchymal cells. Its occurrence activates the mesenchymal phenotypes, which is related to enhanced migratory capacity, invasiveness, and the process increases the production of extracellular matrix components [16, 48]. As a result, LECs lose their normal structure, transdifferentiate into elongated spindle-shaped myofibroblasts, and migrate across the lens capsule, causing cataract formation [48]. Zhang et al. [10] observed decreased cell proliferation and mobility upon specifically inhibiting mTOR in LECs with siRNA. Besides, the function of mTORC1 was decreased, while the EMT level on LECs was inhibited significantly. Furthermore, Meng et al. pointed out that activation of the PI3K/Akt/mTOR axis initiates the process of EMT in human lens epithelial B-3 cells [49]. The study demonstrated that activation of the mTOR pathway with the phosphorylation EIF4EBP promotes the development of cataract, which was consistent with our results. [49].

On the other hand, autophagy is an important factor in regulating cataract evolution. Autophagy is a highly conserved process that involves the degradation of senescent macromolecules and organelles of LECs to maintain lens transparency [49]. Autophagy is the early response to the internal environment disturbance induced by oxidative stress, like starvation, hypoxia, deficiency of growth factors and over-activation. When encountered with stress, autophagy gets up-regulated, making LECs adapt to a new balance. After achieving homeostasis, the autophagy level decreases [9]. Similarly, a previous study observed that amino acid deprivation induces the activation of autophagy, and the process is regulated by mTORC1[50]. Ping and collages[9] reported that ablation of Gja8b in zebrafish causes severe defects in organelle degradation, inducing defective autophagy in LECs and inhibiting the degradation of senescent macromolecules, leading to cataract formation. After being treated with rapamycin, autophagy was promoted in LECs in a dose-dependent manner causing mitigation of cataract. Several conjectures exist for regulating proteins related to EIF4EBP and mTORC1 to control autophagy. The transcription factor FOXO activates EIF4EBP to active autophagy/lysosome system in reducing muscle aging in Drosophila. This system can degrade damaged protein to reduce the accumulation of metabolic stressors [51].

Except autophagy and EMT respective functions in the formation of cataract, they are also reported to interact with each other. For instance, autophagy induction could promote EMT in cardiac fibrotic disease [52], whereas autophagy could contribute to the attenuation of EMT in chronic renal fibrosis[53]. Sun et al. [54] discovered that autophagy inhibition attenuates the EMT process in New Zealand white rabbit LECs, triggered by the TGF-β2/Smad signaling pathway. Therefore, part from autophagy and EMT respective functions in formation of cataract, their interaction may play a critical role in the formation process of cataract. Therefore, their interactions need to be studied further.

In the current study, we observed that EIF4EBP was positively associated with cataract. Whereas RP-S6K, EIF-4E, EIF-4G, and EIF-4A were not significantly associated with cataract. Similar to our study, Zhang et al. [55] compared RNA from age-related cataracts and normal lens epithelia using the semiquantitative RT-PCR method and observed no difference between cataract and normal LECs on EIF-4E levels. We speculate that these proteins may be involved in other signaling pathways. RP-S6k exists in different signal pathways, while S6K/PP1α/B-Raf can activate MAPK in PI3K/AKT signaling in regulating prostate cancer cell migration and invasion[56]. Besides, the EIF-4F complex can also be regulated by protein, except for the classical mTOR related molecule, like PDCD4[57]. PDCD4 is a tumor suppressor and can bind with EIF-4A to limit the available content to form EIF-4F. EIF-4F components can be regulated apart from EIF4EBP, promyelocytic leukemia protein (PML) has been associated with inhibiting the mRNA nuclear export role of EIF-4E [58]; EIF4A3 can combine with RNA-binding motif 8A (RBM8A), Mago homolog (MAGOH), and other auxiliary proteins, and form an exon junction complex (EJC) to regulate gene expression, including mRNA splicing, translation, and degradation [59]; EIF4G can initiate cap-independent translation with the presence of an internal ribosomal entry site (IRES) instead of depending on cap-binding protein eIF4E [60]. In conclusion, according to this MR study, the plasma concentration of mTORC1-depended EIF4EBP promotes the onsetting of cataract on genetic level. Its regulating function may be governed through the mTORC1/EIF4EBP axis. However, we speculate that other potential signaling pathways may regulate these proteins and affect the concentration of RP-S6K, EIF4F components in plasma. Of note, we could not detect the association between cataract and RP-S6K, EIF4F components. Therefore, our study shows that the EIF4EBP is a vital protein associated with cataract. However, further research is required to explore the mTORC1/EIF4EBP axis, the downstream protein of this signaling including RP-S6K and EIF4F components, in the process of cataract formation.

There were some limitations to this present study. Firstly, considering the data source from the GWAS meta-analyses statistic data of mTOR‑dependent circulating protein and cataract were restricted in individuals with European ancestry, we are unclear whether this finding can be applied to other populations. Thus, more sources of data are required to detect the effect on another ethnicity. Secondly, although sensitivity analysis results showed no evidence of unbalanced pleiotropy, part of balanced pleiotropy may exist. Thirdly, more detailed information on the subtype of cataracts has not been available until now. Therefore, further research on the association between mTOR-dependent protein level and subtype of cataracts is required. Fourthly, some researchers indicated that the GWAS only incorporated 3301 individuals because of the expensive measuring technology of plasma protein[31]. Thus, the database cannot detect as many genome-wide significant genetic variants as possible. A more relaxed IV selection threshold might balance between feasibility of the study and statistical power. Additionally, some scholars chose the IV selection threshold to be *P*<5e^− 6^ rather than the commonly used GWAS significance level *P*<5e^− 8^ to obtain sufficient SNPs and to estimate more accurate results [29–31]. Therefore, we set a more relaxed IV selection threshold to be *P*<5e^− 6^ rather than the commonly used GWAS significance level of *P*<5e^− 8^. Fifthly, there are three isoforms for EIF4EBP, including EIF4EBP1, EIF4EBP2 and EIF4EBP3[61]. EIF4EBP1 is abundant in adipose tissue, pancreas, and skeletal muscle, EIF4EBP2 is ubiquitously expressed[62], and EIF4EBP3 distributes highest in skeletal muscle, heart, kidney, and pancreas[63]. Compared with EIF4EBP1, EIF4EBP2 gets phosphorylated at lesser residues[61], has a binding preference with EIF4F [61], and has a more effective blockade by rapamycin[64]. Considering the wide distribution and function of EIF4EBP2, it might play a more critical role in the cataract formation than other two isoforms. At the same time, due to the limitation of the database, we could only incorporate EIF4EBP2 in this study. Further research is needed to test the interaction and regulation between these isoforms. Lastly, although this study provided a credible signaling pathway in the pathogenesis of cataracts, more experimental laboratory data is warranted to verify its feasibility.

## Conclusion

This unbiased two-sample MR study supports the causal positivity association between circulating EIF4EBP levels and cataract formation. Furthermore, using the *in-vitro* and *in-vivo* data of the previous studies, our results suggested the critical role of the mTORC1/EIF4EBP axis in the mechanism of cataract, regardless of cataract subtypes. However, further studies are required to verify this pathway to explore the detailed functional relevance and downstream proteins of the axis and to further investigate its clinical utility. Nevertheless, the finding of this study will provide the basis for a more efficient pharmacological target in the prevention and treatment of cataracts.

## Electronic supplementary material

Below is the link to the electronic supplementary material.

Supplementary Material 1

Supplementary Material 2

Supplementary Material 3

## Data Availability

All data used for this study are publicly available and their original studies are cited from (https://www.phpc.cam.ac.uk/ceu/proteins/ and https://r5.finngen.fi/).

## Abbreviations

mTOR: mechanistic target of rapamycin
EMT: epithelial-mesenchymal transition
mTORC1: mechanistic target of rapamycin complex1
mTORC2: mechanistic target of rapamycin complex2
PR-S6K: ribosomal protein S6K kinase
EIF4EBP: eukaryotic initiation factor 4E-binding protein
EIF-4G: eukaryotic initiation factor 4G
EIF-4F: eukaryotic initiation factor 4F
EIF-4B: eukaryotic initiation factor 4B
EIF-4A: eukaryotic translation factor 4A
EIF-4A: eukaryotic translation factor 4A
SNP: single-nucleotide polymorphisms
GWAS: genome-wide association studies
LEC: lens epithelial cell
PI3K: phosphatidylinositol 3-kinase
