# Supplementary material for: Association of mTORC1‑dependent circulating protein levels with cataract formation: a mendelian randomization study
Source: BMC Genomics. 2022 Oct 21;23:719. doi: 10.1186/s12864-022-08925-7 (PMC9587558; doi:10.1186/s12864-022-08925-7)
Supplement: Supplementary file 1 — Supplementary Material 1 [file 12864_2022_8925_MOESM1_ESM.docx]

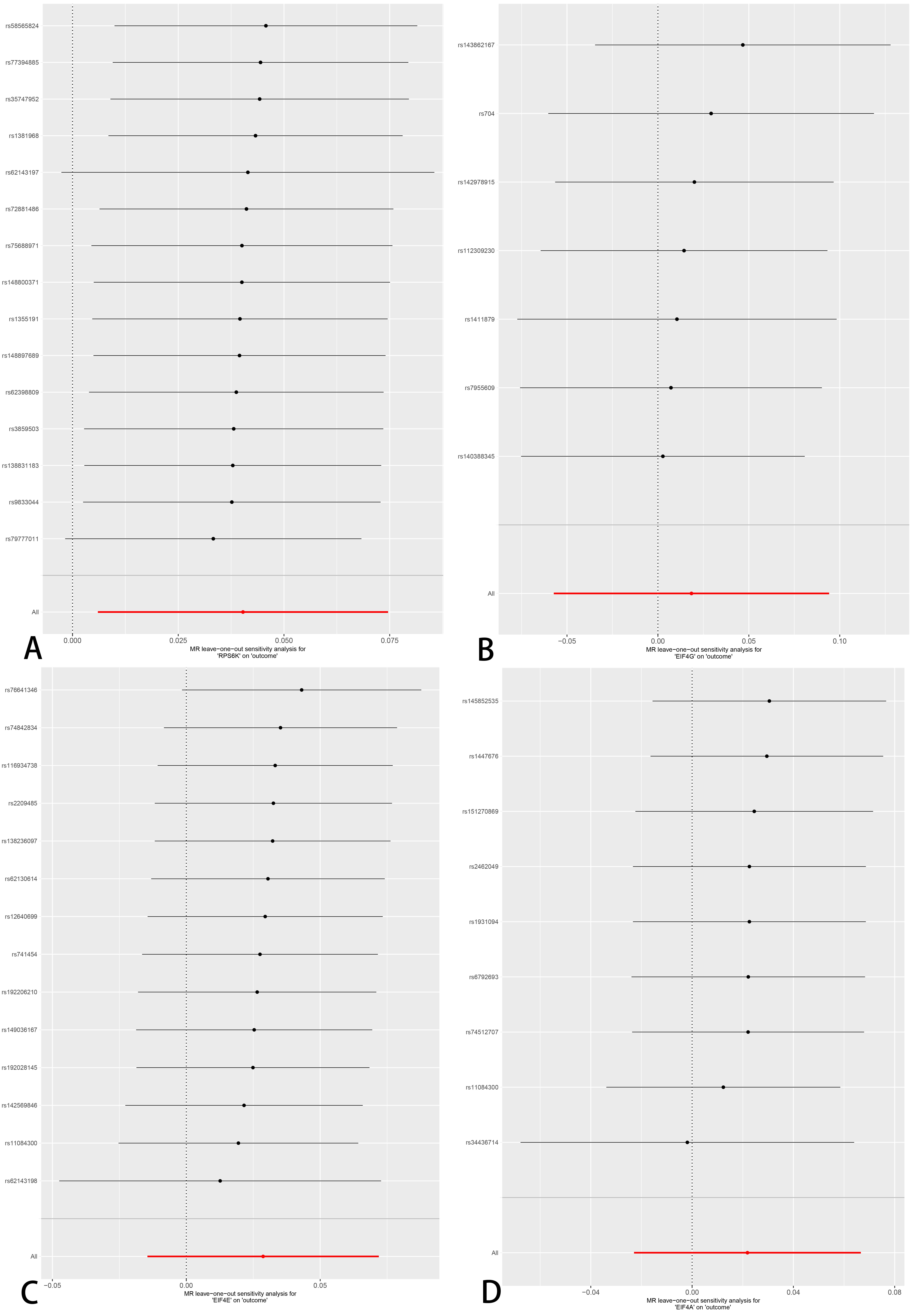


**Supplementary Figure 2 leave-one-out analysis for the estimates of RP-S6K, EIF-4G, EIF-4E, and EIF-4A on cataract.**

Leave-one-out result of (A) RP-S6K, (B)EIF-4G, (C) EIF-4E, and (D) EIF-4A. The leave-one-out result shows that after excluding one SNP, the result of the MR estimate was almost stable, which further verified the robustness of MR results. RP-S6K, ribosomal protein S6K kinase; EIF-4G, translation initiation factor 4G; EIF-4E, translation initiation factor 4E; EIF-4A, translation initiation factor 4A; MR, Mendelian randomization
