# Supplementary material for: Association of mTORC1‑dependent circulating protein levels with cataract formation: a mendelian randomization study
Source: BMC Genomics. 2022 Oct 21;23:719. doi: 10.1186/s12864-022-08925-7 (PMC9587558; doi:10.1186/s12864-022-08925-7)
Supplement: Supplementary file 2 — Supplementary Material 2 [file 12864_2022_8925_MOESM2_ESM.docx]

**Supplementary Table 1 Mendelian Randomization estimate of mTORC1 related protein with outcome SNPs**

|  | SNP | Effect  allele | Palindromic | Beta  Exposure | SE  Exposure | *P-value*  Exposure | Beta  Outcome | SE  Outcome | *P-value*  Outcome |
| --- | --- | --- | --- | --- | --- | --- | --- | --- | --- |
| **RP-S6K** | | | | | | | | | |
| 1 | rs1355191 | C | FALSE | -0.125 | 0.026 | 1.95×10^-6^ | -0.008 | 0.012 | 0.521 |
| 2 | rs1381968 | T | FALSE | -0.346 | 0.075 | 3.55×10^-6^ | 0.023 | 0.037 | 0.532 |
| 3 | rs138831183 | G | FALSE | 0.518 | 0.107 | 1.45×10^-6^ | 0.048 | 0.043 | 0.267 |
| 4 | rs148800371 | T | FALSE | -0.164 | 0.033 | 5.50×10^-7^ | -0.008 | 0.014 | 0.594 |
| 5 | rs148897689 | G | FALSE | 0.450 | 0.094 | 1.91×10^-6^ | 0.046 | 0.069 | 0.504 |
| 6 | rs17412698 | G | TRUE | -0.239 | 0.052 | 4.27×10^-6^ | 0.074 | 0.029 | 0.009 |
| 7 | rs35747952 | T | FALSE | -0.374 | 0.081 | 3.89×10^-6^ | 0.011 | 0.028 | 0.709 |
| 8 | rs3859503 | A | FALSE | 0.189 | 0.033 | 1.35×10^-8^ | 0.014 | 0.014 | 0.297 |
| 9 | rs58565824 | C | FALSE | 0.461 | 0.094 | 8.91×10^-7^ | -0.010 | 0.028 | 0.730 |
| 10 | rs62143197 | G | FALSE | 0.535 | 0.029 | 8.13×10^-76^ | 0.021 | 0.015 | 0.166 |
| 11 | rs62398809 | A | FALSE | -0.113 | 0.025 | 4.90×10^-6^ | -0.010 | 0.012 | 0.368 |
| 12 | rs72881486 | G | FALSE | 0.363 | 0.075 | 1.07×10^-6^ | 0.003 | 0.041 | 0.946 |
| 13 | rs74353857 | G | TRUE | -0.252 | 0.053 | 1.51×10^-6^ | 0.004 | 0.019 | 0.838 |
| 14 | rs75688971 | T | FALSE | 0.418 | 0.084 | 5.75×10^-7^ | 0.018 | 0.028 | 0.509 |
| 15 | rs77394885 | A | FALSE | 0.529 | 0.112 | 2.40×10^-6^ | -0.037 | 0.049 | 0.449 |
| 16 | rs79549584 | A | TRUE | -0.280 | 0.042 | 2.75×10^-11^ | 0.015 | 0.021 | 0.455 |
| 17 | rs79777011 | C | FALSE | 0.163 | 0.035 | 2.29×10^-6^ | 0.034 | 0.014 | 0.017 |
| 18 | rs9833044 | C | FALSE | -0.319 | 0.064 | 5.13×10^-7^ | -0.029 | 0.025 | 0.248 |
| **EIF4EBP** | | | | | | | | | |
| 1 | rs10733789 | T | FALSE | 0.125 | 0.027 | 4.07×10^-6^ | -1.00×10^-4^ | 0.013 | 0.865 |
| 2 | rs10864412 | G | FALSE | -0.133 | 0.025 | 1.58×10^-7^ | -0.021 | 0.012 | 0.882 |
| 3 | rs113664570 | C | FALSE | 0.267 | 0.058 | 4.90×10^-6^ | -1.00×10^-4^ | 0.042 | 0.454 |
| 4 | rs11708375 | G | TRUE | 0.158 | 0.034 | 3.39×10^-6^ | -0.016 | 0.015 | 0.991 |
| 5 | rs17003636 | T | FALSE | 0.461 | 0.098 | 2.88×10^-6^ | 0.040 | 0.031 | 0.0771 |
| 6 | rs186490680 | C | FALSE | -0.451 | 0.095 | 2.09×10^-6^ | 0.013 | 0.086 | 0.999 |
| 7 | rs2745108 | T | FALSE | -0.193 | 0.040 | 1.58×10^-6^ | -0.067 | 0.026 | 0.301 |
| 8 | rs6993770 | A | TRUE | -0.139 | 0.027 | 3.89×10^-7^ | -0.022 | 0.014 | 0.251 |
| 9 | rs72743058 | G | FALSE | -0.384 | 0.083 | 3.47×10^-6^ | -0.070 | 0.042 | 0.878 |
| 10 | rs72806714 | G | TRUE | -0.138 | 0.030 | 3.09×10^-6^ | -0.006 | 0.014 | 0.011 |
| 11 | rs76802510 | C | FALSE | -0.268 | 0.050 | 6.46×10^-8^ | -0.025 | 0.021 | 0.121 |
|  | SNP | Effect  allele | Palindromic | Beta  Exposure | SE  Exposure | P value  Exposure | Beta  Outcome | SE  Outcome | P value  Outcome |
| 12 | rs79613514 | T | FALSE | 0.341 | 0.074 | 3.89×10^-6^ | -0.007 | 0.026 | 0.095 |
| 13 | rs79943794 | C | FALSE | -0.357 | 0.077 | 3.16×10^-6^ | -0.042 | 0.033 | 0.679 |
| **EIF-4G** | | | | | | | | | |
| 1 | rs112309230 | T | FALSE | -0.575 | 0.116 | 6.92×10^-7^ | -0.038 | 0.079 | 0.631 |
| 2 | rs140388345 | G | FALSE | 0.559 | 0.120 | 3.31×10^-6^ | 0.153 | 0.090 | 0.087 |
| 3 | rs1411879 | G | FALSE | 0.409 | 0.089 | 4.27×10^-6^ | 0.017 | 0.031 | 0.585 |
| 4 | rs142978915 | T | FALSE | -0.406 | 0.089 | 4.57×10^-6^ | 0.021 | 0.105 | 0.840 |
| 5 | rs143862167 | C | FALSE | -0.204 | 0.044 | 3.55×10^-6^ | 0.034 | 0.022 | 0.115 |
| 6 | rs6993770 | A | TRUE | -0.130 | 0.027 | 2.14×10^-6^ | -0.022 | 0.014 | 0.121 |
| 7 | rs704 | G | FALSE | 0.162 | 0.025 | 4.27×10^-11^ | -0.001 | 0.012 | 0.907 |
| 8 | rs7955609 | G | FALSE | 0.125 | 0.026 | 2.00×10^-6^ | 0.009 | 0.012 | 0.432 |
| **EIF-4E** | | | | | | | | | |
| 1 | rs11084300 | C | FALSE | 0.159 | 0.027 | 5.75×10^-9^ | 0.024 | 0.013 | 0.067 |
| 2 | rs116934738 | T | FALSE | 0.376 | 0.077 | 1.05×10^-6^ | -0.042 | 0.047 | 0.374 |
| 3 | rs12640699 | C | FALSE | 0.157 | 0.034 | 4.07×10^-6^ | 8.00×10^-4^ | 0.020 | 0.969 |
| 4 | rs138236097 | G | FALSE | -0.267 | 0.058 | 4.47×10^-6^ | 0.017 | 0.031 | 0.575 |
| 5 | rs142569846 | G | FALSE | 0.230 | 0.047 | 8.71×10^-7^ | 0.038 | 0.023 | 0.098 |
| 6 | rs149036167 | T | FALSE | -0.517 | 0.108 | 1.70×10^-6^ | -0.056 | 0.057 | 0.324 |
| 7 | rs192028145 | A | FALSE | -0.530 | 0.103 | 2.40×10^-7^ | -0.153 | 0.097 | 0.116 |
| 8 | rs192206210 | T | FALSE | 0.585 | 0.121 | 1.41×10^-6^ | 0.038 | 0.054 | 0.479 |
| 9 | rs2209485 | C | FALSE | 0.164 | 0.034 | 1.51×10^-6^ | -0.008 | 0.016 | 0.645 |
| 10 | rs531476076 | G | TRUE | 0.567 | 0.118 | 1.41×10^-6^ | 0.066 | 0.045 | 0.147 |
| 11 | rs62130614 | G | FALSE | 0.461 | 0.010 | 3.98×10^-6^ | -0.030 | 0.074 | 0.682 |
| 12 | rs62143198 | G | FALSE | 0.467 | 0.030 | 6.76×10^-6^ | 0.021 | 0.015 | 0.149 |
| 13 | rs741454 | T | FALSE | 0.143 | 0.031 | 4.17×10^-6^ | 0.008 | 0.016 | 0.606 |
| 14 | rs74842834 | G | FALSE | 0.444 | 0.095 | 2.69×10^-6^ | -0.174 | 0.079 | 0.028 |
| 15 | rs76641346 | G | FALSE | 0.305 | 0.063 | 1.26×10^-6^ | -0.054 | 0.026 | 0.040 |
| 16 | rs7862784 | G | TRUE | -0.123 | 0.026 | 2.88×10^-6^ | -0.012 | 0.012 | 0.321 |
| **EIF-4A** | | | | | | | | | |
| 1 | rs11084300 | C | FALSE | 0.143 | 0.027 | 1.48×10^-7^ | 0.024 | 0.013 | 0.067 |
| 2 | rs1447676 | C | FALSE | -0.118 | 0.026 | 4.37×10^-6^ | 0.015 | 0.012 | 0.226 |
| 3 | rs145852535 | A | FALSE | 0.521 | 0.112 | 3.16×10^-6^ | -0.063 | 0.050 | 0.208 |
| 4 | rs151270869 | A | FALSE | -0.454 | 0.094 | 1.29×10^-6^ | 0.003 | 0.035 | 0.944 |
|  | SNP | Effect  allele | Palindromic | Beta  Exposure | SE  Exposure | P value  Exposure | Beta  Outcome | SE  Outcome | P value  Outcome |
| 5 | rs1931094 | G | FALSE | 0.116 | 0.025 | 4.27×10^-6^ | 9.00×10^-4^ | 0.012 | 0.938 |
| 6 | rs2462049 | G | FALSE | 0.116 | 0.025 | 3.31×10^-6^ | 9.00×10^-4^ | 0.012 | 0.940 |
| 7 | rs34436714 | C | FALSE | 0.469 | 0.029 | 2.00×10^-6^ | 0.020 | 0.015 | 0.173 |
| 8 | rs3846325 | T | TRUE | 0.130 | 0.028 | 3.39×10^-6^ | 0.020 | 0.013 | 0.131 |
| 9 | rs6792693 | A | FALSE | 0.134 | 0.029 | 2.82×10^-6^ | 0.002 | 0.013 | 0.865 |
| 10 | rs74512707 | G | FALSE | 0.179 | 0.039 | 3.55×10^-6^ | 0.003 | 0.019 | 0.882 |
| 11 | rs79549584 | A | TRUE | -0.216 | 0.042 | 3.09×10^-6^ | 0.015 | 0.021 | 0.454 |

Abbreviations: SNP, single nucleotide polymorphism; RP-S6K, ribosomal protein S6K kinase; EIF4EBP, eukaryotic initiation factor 4E-binding protein; EIF-4G, translation initiation factor 4G; EIF-4E, translation initiation factor 4E; EIF-4A, translation initiation factor 4A.
