# Supplementary material for: Association of mTORC1‑dependent circulating protein levels with cataract formation: a mendelian randomization study
Source: BMC Genomics. 2022 Oct 21;23:719. doi: 10.1186/s12864-022-08925-7 (PMC9587558; doi:10.1186/s12864-022-08925-7)
Supplement: Supplementary file 3 — Supplementary Material 3 [file 12864_2022_8925_MOESM3_ESM.docx]

**Supplementary Figure 1 funnel plots of the MR study**

Funnel plots of (A) RP-S6K, (B) EIF4EBP, (C) EIF-4G, (D) EIF-4E, and (E) EIF-4A. All funnel plots are almost symmetrical, which means no evidence of pleiotropy. RP-S6K, ribosomal protein S6K kinase; EIF4EBP, eukaryotic initiation factor 4E-binding protein; EIF-4G, translation initiation factor 4G; EIF-4E, translation initiation factor 4E; EIF-4A, translation initiation factor 4A
